# The Potential Role of Mycotoxins as a Contributor to Stunting in the SHINE Trial

**DOI:** 10.1093/cid/civ849

**Published:** 2015-11-11

**Authors:** Laura E. Smith, Andrew J. Prendergast, Paul C. Turner, Mduduzi N. N. Mbuya, Kuda Mutasa, George Kembo, Rebecca J. Stoltzfus

**Affiliations:** 1 Division of Nutritional Sciences, Cornell University, Ithaca, New York; 2 Blizard Institute, Queen Mary University of London, United Kingdom; 3 Zvitambo Institute for Maternal and Child Health Research, Harare, Zimbabwe; 4 Maryland Institute for Applied Environmental Health, School of Public Health, University of Maryland, College Park; 5 Food and Nutrition Council, Harare, Zimbabwe

**Keywords:** stunting, aflatoxin, fumonisin, deoxynivalenol, mycotoxin

## Abstract

Children in developing countries experience multiple exposures that are harmful to their growth and development. An emerging concern is frequent exposure to mycotoxins that contaminate a wide range of staple foods, including maize and groundnuts. Three mycotoxins are suspected to contribute to poor child health and development: aflatoxin, fumonisin, and deoxynivalenol. We summarize the evidence that mycotoxin exposure is associated with stunting, and propose that the causal pathway may be through environmental enteric dysfunction (EED) and disturbance of the insulin-like growth factor 1 (IGF-1) axis. The objectives of this substudy are to assess the relationship between agricultural and harvest practices and mycotoxin exposure; to evaluate associations between mycotoxin exposure and child stunting; and to investigate EED as a potential pathway linking mycotoxin exposure to child stunting, to inform potential areas for intervention.

Children in developing countries experience multiple exposures that are harmful to their health, growth, and development, particularly during the critical first 1000 days, including nutrient deficiencies, recurrent infections, and exposure to human and animal feces. An emerging additional concern is frequent exposure to mycotoxins [1], which are toxic secondary metabolites of fungi that contaminate a wide range of staple foods such as maize and groundnuts. Recent developments in biomarkers now enable exposure measurement to 3 mycotoxins that may be important causes of poor child health and development: aflatoxin, fumonisin, and deoxynivalenol [1]. This article describes the rationale and methods for the investigation of the potential role of mycotoxins as a contributor to stunting among children enrolled in the Sanitation Hygiene Infant Nutrition Efficacy (SHINE) trial in Zimbabwe.

The SHINE trial will evaluate the independent and combined effects of an integrated water, sanitation, and hygiene (WASH) intervention and an infant and young child feeding (IYCF) intervention on stunting and anemia in rural Zimbabwean infants through 18 months of age [2]. We hypothesize that protection from fecal microbes will prevent environmental enteric dysfunction (EED) [3] and systemic inflammation and thereby remove constraints on the growth hormone insulin-like growth factor 1 (IGF-1) axis that governs linear growth [4, 5]. It is also possible that exposure to mycotoxins pre- and postnatally contributes to stunting, in part through common pathways of EED and IGF-1 suppression [1, 6].

## EVIDENCE THAT MYCOTOXINS ARE ASSOCIATED WITH CHILD STUNTING

It is estimated that approximately 4.5 billion people, predominantly those living in developing countries, are at risk of exposure to the dietary mycotoxin family of aflatoxins (AFs), with people in some regions experiencing chronic exposure at high levels [7]. AFs have been most widely studied and demonstrated as a cause of liver cancer [8]. AFs additionally inhibit protein synthesis and are cytotoxic, teratogenic, and immunotoxic [8]. Epidemiologic evidence from multiple countries documents AF exposure in pregnant women, infant cord blood, and young children [9–18], suggesting that exposure to AF is widespread during early life. Gong et al [16] found that serum aflatoxin-albumin (AF-alb) adducts were associated with stunting in children (aged 9–60 months) in rural Benin and Togo, and demonstrated a significant dose-response relationship with height-for-age and weight-for-age *z* scores. In a subsequent 8-month longitudinal study in rural Benin, infants (aged 16–37 months at recruitment) were on average 1.7 cm shorter in the highest quartile of AF-alb compared with the lowest quartile of exposure [19]. In a separate study from The Gambia, maternal exposure during pregnancy was strongly inversely associated with infant growth velocity during the first 12 months of life, with a predicted 0.8-kg increase in weight and 2-cm increase in height, if measures of maternal AF exposure (AF-alb) were reduced from 110 pg/mg to 10 pg/mg [18]. The range of exposure measures in these 3 regions was 3–1080 pg/mg [17, 18, 20]. This height difference is meaningful and large in public health terms, compared to the effects of efficacious nutrition interventions [20].

Two other mycotoxins, fumonisin (FUM) and deoxynivalenol (DON), also often contaminate staple foods, and both have plausible links to impaired infant growth. FUM inhibits ceramide synthase, an enzyme essential to sphingolipid metabolism [21]. Complex sphingolipids are integral to cell membrane integrity, and disturbance in this biosynthetic pathway could affect intestinal epithelial cell viability and proliferation, modify cytokine production, and modulate intestinal barrier function. A urinary biomarker for FUM (urinary fumonisin B1 [FB1]) was recently reported [22], and the first population-based longitudinal study in Tanzanian infants (aged 6–14 months at recruitment) found a significant association between urinary FB1 and growth faltering [23]. In this study from 3 regions of Tanzania, 98% of infants (157/160) were exposed to FUM and 67% (98/157) were exposed to AF [23]. AF exposures were lower than those in the Beninese and Gambian studies, and the observed inverse relationship with growth faltering did not reach statistical significance [23].

It is plausible that DON has a negative effect on growth because of decreased food intake and reduced weight gain, which has been observed in animal studies [6]. Rotter et al [24] found that pigs fed grain contaminated with DON had 20% lower feed intake and 13% lower weight gain than the control group and suggested that DON induces feed refusal in pigs. In a recent study, Amuzie and Pestka [25] found that DON intake in mice induced a decrease in circulating levels of IGF-1, the predominant mediator of growth hormone activity, and hepatic insulin-like growth factor acid-labile subunit, which forms a complex with circulating IGF-1. The effects of DON exposure on child growth have not yet been studied; however, in the same group of Tanzanian infants (mentioned above), 51% had detectable levels of DON [26]. In another study of pregnant Egyptian women, AF and DON biomarkers were concurrently found in 41% of the women [27]. Recent biomarker surveys from Cameroon and Nigeria support the concept of frequent coexposures to mixtures of mycotoxins [28, 29]; thus, exposure to 1 or more mycotoxins during pregnancy and infancy is likely common and may contribute to the complex etiology of stunting.

## A HYPOTHESIZED PATHWAY THROUGH EED

All 3 mycotoxins may plausibly contribute to stunting through EED [3], a subclinical condition of the small intestine that impairs nutrient absorption and causes systemic immune activation [2, 3]. EED has most commonly been attributed to environmental contamination with fecal bacteria in disadvantaged settings, where sanitation, hygiene practices, and drinking water quality are frequently poor [2]. However, there are multiple overlapping causes of enteropathies in developing countries [4], and the role of mycotoxins in mediating enteropathy has received little attention to date. Although the mycotoxins described here have distinct actions, they all mediate intestinal damage in experimental animal models through (1) inhibition of protein synthesis (AF, DON); (2) increased local and systemic proinflammatory cytokines (AF, DON); (3) inhibition of ceramide synthase (FUM) (reviewed in [6]); and (4) tight junction protein expression (DON, FUM) [30]. AF and DON may also directly cause immunomodulation toward an inflammatory state, potentially interfering with the IGF-1 axis [31–34]. In vitro models additionally support a role for mycotoxins disrupting intestinal cell monolayer integrity (AF) [35]. AF, FUM, and DON may therefore share a convergent pathway in which mucosal damage can lead to impaired nutrient absorption and/or increased intestinal permeability, pathology that resembles the changes seen in EED [6].

## MYCOTOXIN INVESTIGATIONS IN THE SHINE TRIAL

The overall objective of this substudy is to describe the risk factors contributing to mycotoxin exposure and to assess the potential role of mycotoxin exposure in the pathogenesis of stunting. Our overarching hypothesis is that AF exposure, alone or in combination with FUM and DON, contributes to EED and is an important cause of child stunting. Specifically, this substudy will assess the relationship between agricultural and harvest practices and mycotoxin exposure; assess the relationship between mycotoxin exposure and child stunting; and investigate EED as a potential pathway linking mycotoxin exposure to child stunting.

## INVESTIGATION OF AGRICULTURAL AND HARVEST PRACTICES ASSOCIATED WITH MYCOTOXIN EXPOSURE

Pre- and postharvest crop management has a significant influence on the accumulation of AF in maize and groundnuts [36, 37]. Smallholder farming practices are shaped by the ecological and social context, and information is scarce about farmers' practices and how their decisions affect household-level exposure to mycotoxins. With a view to increasing local knowledge and informing future preventive intervention strategies, we seek to understand the drivers of AF exposure in rural Zimbabwe.

We found no precedent in the literature for a survey module designed to assess mycotoxin risk according to agricultural practices by smallholder farmers. We therefore designed a survey module comprising closed-ended questions regarding pre- and postharvest practices relevant to maize and groundnuts (Table 1 in Supplementary Appendix). This module was translated, pilot-tested among rural households, and revised for clarity before implementation within the SHINE baseline survey. The baseline survey includes additional modules related to household wealth, land ownership, food security, infant feeding practices, and dietary diversity, which complement the mycotoxin risk module.

These data will be analyzed to provide a description of relevant agricultural practices, including a summary mycotoxin risk indicator. In addition, we will describe the association between mycotoxin risk and household characteristics (eg, wealth, land ownership), geographic locale, season, year, and rainfall. Last, we will ascertain the extent to which overall mycotoxin risk (measured as a summary score) and/or specific practices are associated with biomarkers of mycotoxin exposure in SHINE mothers and infants.

## ASSESSING THE RELATIONSHIP BETWEEN MYCOTOXIN EXPOSURE AND CHILD STUNTING

We will assess the longitudinal relationship between infant mycotoxin exposure and growth from 0–18 months, in human immunodeficiency virus (HIV)–uninfected mother–infant dyads. Eligible infants will include all HIV-unexposed infants born from November 2014 through September 2015 (N = 1000), and will include approximately equal numbers of infants from the WASH and IYCF arms of the SHINE trial. Because HIV exposure affects postnatal growth independent of mycotoxins [38], we will exclude infants born to HIV-infected mothers to avoid this source of variation. Maternal HIV testing is undertaken at baseline and at 32 weeks of gestation; women testing negative at these time-points will be included in this substudy. There is little basis for sample size calculations; however, previous studies reporting positive associations between AF or FUM biomarkers and child growth have employed sample sizes ranging from around 100 to several hundred [16, 18, 23].

Serum AF-alb and urinary FUM and DON will be measured using liquid chromatography–mass spectrometry [39, 40]; these parent mycotoxins and metabolites have a quantitative relationship with dietary intake of the toxin and therefore serve as useful quantitative exposure biomarkers [22, 41, 42]. AF-alb adduct will be measured in all mothers at enrollment (median, 14 weeks’ gestation) and around 32 weeks’ gestation, and in infants at 12 and 18 months of age. All 3 mycotoxins (AF-alb adduct, urinary FB1, and urinary DON) will be measured in a subgroup of 200 infants at 6, 12, and 18 months. These multiple exposure analyses will be exploratory in nature but will be important findings because only 2 studies to date have evaluated FUM and DON biomarkers in African infants [23, 26].

Our first step in the analysis will be to describe the prevalence and severity of mycotoxin exposures in Zimbabwean infants by age and by season. In the 200 infants with multiple mycotoxin assessments, we will describe the frequency, concentration, and covariance of the 3 exposures. We will test the association with stunting using multivariate regression models, with linear growth as the outcome variable and mycotoxin exposure as the independent variable. Linear growth will be modeled both as a continuous variable (attained height-for-age *z* score) using logistic regression and as a dichotomous variable (stunted vs not stunted), and using ordinal logistic regression with 3 levels (nonstunted, moderately stunted, and severely stunted). We will explore whether the mycotoxin–stunting relationships are independent of the SHINE WASH intervention, by examining these models for the WASH and non-WASH groups separately.

## INVESTIGATION OF EED AS A CAUSAL PATHWAY LINKING MYCOTOXINS TO STUNTING

We hypothesize that mycotoxin exposure will be associated with EED and that this is an important causal pathway through which mycotoxins mediate stunting. The biomarkers used in this analysis will evaluate multiple domains of EED, as described elsewhere [2, 3]. Our statistical approach will be the same as that described above for the outcome of stunting. We will additionally test whether EED, diarrhea, and decreased appetite mediate the association between mycotoxins and stunting [43].

We have estimated our power using the Vittinghoff method for sample size calculation for evaluating mediation [44]. With 1000 infants, we will be able to detect an effect size of 30% of 1 standard deviation, with lactulose-mannitol ratio [3] as our primary mediator.

## DISCUSSION

In summary, the analyses described in this article will provide foundations for future research by exploring the potential mechanisms linking mycotoxin exposure to child stunting, and will provide knowledge about relevant agricultural practices that could be targeted in the development of interventions to reduce mycotoxin exposure in vulnerable populations. This study will provide data on levels and frequencies of multiple mycotoxin exposures in our study population, thereby capturing typical exposure patterns within a region. The longitudinal assessment of mycotoxins will allow us to understand the effect of multiple interacting exposures in a well-characterized cohort of infants. To our knowledge, only 1 previous study has investigated multiple mycotoxin exposure in infants longitudinally, and no study has assessed the combined effects of multiple mycotoxins on stunting while testing a specific causal pathway through EED and the IGF-1 axis. Our additional data on agricultural and harvest practices associated with mycotoxin exposure will allow the direct translation of this work into the development of interventions targeting modifiable behaviors to prevent future exposure.

## Supplementary Data

Supplementary materials are available at *Clinical Infectious Diseases* online (http://cid.oxfordjournals.org). Supplementary materials consist of data provided by the author that are published to benefit the reader. The posted materials are not copyedited. The contents of all supplementary data are the sole responsibility of the authors. Questions or messages regarding errors should be addressed to the author.
